# Experience of healthcare discrimination reported by individuals with a history of gynecologic cancer in the *All of Us* research program

**DOI:** 10.1016/j.gore.2025.101723

**Published:** 2025-03-16

**Authors:** Allison C. Dona, Patricia I. Jewett, Sarah Davidson, Deanna Teoh, Rachel I. Vogel

**Affiliations:** a School of Medicine, University of Minnesota, Minneapolis, MN, USA; b Division of Epidemiology and Community Health, School of Public Health, University of Minnesota, Minneapolis, MN, USA; c Department of Obstetrics, Gynecology and Women’s Health, Division of Gynecologic Oncology, University of Minnesota, Minneapolis, MN, USA; d Department of Medicine, Division of Hematology and Oncology, University of Minnesota, Minneapolis, MN, USA; e Masonic Cancer Center, University of Minnesota, Minneapolis, MN, USA

**Keywords:** Healthcare discrimination, Discrimination, Cervical cancer, Endometrial cancer, Ovarian cancer, Vulvar cancer, Vaginal cancer

## Abstract

**Objective:**

We aimed to describe prevalence of healthcare discrimination in *All of Us* participants with a history of gynecologic cancer and compare patient experiences of discrimination by gynecologic cancer type.

**Methods:**

We analyzed survey data of participants with a history of gynecologic cancer in the *All of Us Research Program*. Cancer status was defined using self-report or electronic health record documentation. Healthcare discrimination was measured by the Discrimination in Medical Settings (DMS) Scale. Potential confounders were self-reported (cigarette use, sexual orientation, gender, race, ethnicity, income, education, and age) and physical measurement (body mass index). We report associations between gynecologic cancer site (cervical, ovarian, uterine, vaginal or vulvar, more than one) and DMS scores using multivariable logistic regression.

**Results:**

Of 2,195 participants, 1,001 had cervical, 579 uterine, 419 ovarian, 30 vaginal/vulvar, and 166 more than one gynecologic cancer. Most (76.5%) reported experiencing any healthcare discrimination, and 45.0% reported frequent healthcare discrimination. Those with a history of cervical cancer (52%, OR: 1.80, 95% CI: 1.42, 2.28) and multiple cancers (47.8%, OR: 1.53, 95% CI: 1.05, 2.21) had higher odds of frequently reporting discrimination than those with a history of ovarian cancer (37.5%) in the unadjusted model. The relationship observed remained but was attenuated in the adjusted model (Cervical OR: 1.35, 95% CI: 1.04, 1.75; Multiple OR: 1.35 95% CI: 0.91, 2.00).

**Conclusions:**

Healthcare discrimination was reported across cancer types. Individuals with a history of cervical or multiple gynecologic malignancies reported frequent healthcare discrimination more often than those with other gynecologic cancers.

## Highlights

• Gynecologic cancer survivors report previous experience of healthcare discrimination.
• Those with a history of cervical or multiple gynecologic cancers more often reported frequent healthcare discrimination.
• Cancer site differences remained after controlling for potential confounders between cancer type and discrimination.

## 1 Introduction

One out of five adults report experiencing discrimination in a healthcare setting and, of those reporting discrimination, 72 % report experiencing discrimination more than once (Nong et al., 2020). Healthcare discrimination does not impact all groups equitably and may be more common among people of color, individuals with large bodies, women, sexual and gender minorities, individuals with worse self-reported health, and those with lower socioeconomic status, although these findings vary by study (Ayhan et al., 2020, Campesino et al., 2012, Caston et al., 2022, Dibble and Connor, 2023, Macintosh et al., 2013, Nong et al., 2020). Healthcare discrimination may also differ by type of disease because some diseases are more stigmatized than others (Best & Arseniev-Koehler, 2023). Perceived healthcare discrimination has been associated with worse patient outcomes and care quality, including psychological distress, depressive symptoms, strained patient-provider communication, new or worsened disability, and less follow-up care (Attanasio et al., 2021, Brooks et al., 2023, Li et al., 2022, Rogers et al., 2015, Sheppard et al., 2014).

Our understanding of healthcare discrimination specific to those diagnosed with cancer is growing. A national United States (US) survey—as part of the Elevating Cancer Equity Initiative from the American Cancer Society Cancer Action Network, the National Comprehensive Cancer Network, and the National Minority Quality Forum—was conducted to explore the experience of patients and oncologists with bias and discrimination in cancer care (Schatz et al., 2022). Among responding oncologists, only about half (54 %) believed that patients were treated unfairly based on their racial and ethnic background, despite documented disparities in healthcare discrimination by race and ethnicity (Garrett et al., 2024, Schatz et al., 2022, Sutton et al., 2021). Additionally, 44 % believed it was possible that their unintentional biases impacted their care delivery, but biases are considered to be universally prevalent in the general population (Schatz et al., 2022). In a separate sample of cancer survivors, 23 % reported healthcare discrimination, with the most common reasons for discrimination including disease status and income/ability to pay (Caston et al., 2022).

Healthcare discrimination may differ by cancer type, and individuals with gynecologic cancer may be at elevated risk. A study of individuals with a history of cancer and financial limitations reported healthcare discrimination by cancer types, and while the sample size was small for each cancer, those with gynecologic cancers had the second highest proportion reporting healthcare discrimination among groups compared; colorectal cancer survivors reported the highest proportion of healthcare discrimination, followed by gynecologic, genitourinary, other, breast, and hematologic cancers (Caston et al., 2022). While not specifically investigated in this context, potential mechanisms causing those with gynecologic cancer to experience a greater burden of healthcare discrimination compared to those with cancers from other organ systems may include gender- and sex-based discrimination and/or stigmatization of sexual and gynecologic health (Bohren et al., 2022, Govender and Penn-Kekana, 2008).

Some individuals with gynecologic cancer may be at greater risk for healthcare discrimination than others. While care is provided by the same physicians and care teams, gynecologic cancer types have varying prevalence, risk factors, and demographic distributions (Crosbie et al., 2022, Stewart et al., 2013, Yi et al., 2021). Cervical cancer is known to be commonly stigmatized because of its association with sexual health and Human Papilloma Virus (HPV) as its causal mechanism (Peterson et al., 2021). While stigma in the context of other types of gynecologic cancer types has been less frequently studied, vaginal and vulvar cancers may be similarly stigmatized because of their association with sexual health and HPV, and uterine/endometrial cancers may be stigmatized because obesity is a strong risk factor (Crosbie et al., 2022, Fruh et al., 2016). It is thus possible that cancer type may be associated with differences in perception, stigma, bias, and discrimination by care teams.

The objective of this study was to examine perceived healthcare discrimination reported by individuals with a history of gynecologic cancer and to investigate differences by gynecologic cancer type. We hypothesized that individuals with cancers that can be caused by barriers to cancer prevention healthcare (e.g. HPV vaccination, screening and removal of precancerous lesions) and cancers associated with commonly stigmatized health conditions, such as cervical cancer and endometrial cancer (Alfaro et al., 2021, Crosbie et al., 2022, Fruh et al., 2016, Peterson et al., 2021, Stewart et al., 2013), would report more frequent experience of healthcare discrimination.

## 2 Methods

### 2.1 Data Source

We analyzed data from the *All of Us Research Program*, a National Institutes of Health dataset that aims to represent more diverse participants than other existing national United States datasets (Tesfaye et al., 2024). The *All of Us Research Program* began in 2015 and, at time of creation of the dataset for this analysis (September 2023), included over 500,000 participants with a combination of survey data, physical measurements, genotyping arrays, electronic health records, Fitbit records, whole genome sequences, and long-read sequences. The study population for this project was comprised of participants with a history of any primary gynecologic cancer who completed the social determinants of health survey, which contained the healthcare discrimination questions. The University of Minnesota Institutional Review Board deemed this study exempt (STUDY00018637). *All of Us* requires authorized users to know and follow Common Rule Principles as defined by The Belmont Report.

### 2.2 Measures

Gynecologic cancer type was the primary exposure of interest for this analysis. Diagnosis of and type of gynecologic cancer was identified using two sources: documentation in the electronic health record on two or more occasions or self-reported gynecologic cancer type from the personal and family health history form. Participants must have had a gynecologic malignancy diagnosis on two or more occasions in their electronic health record to limit misclassification. In the personal and family health history form, participants were asked if they had been diagnosed with cervical, ovarian, or endometrial cancer. Participants were not asked about vulvar or vaginal cancer diagnoses in the personal and family health history survey. Vulvar and vaginal cancers were ultimately combined because of small sample sizes. Participants identified with primary gynecologic malignancies of multiple primary organ sites were categorized as “More than One”.

The primary outcome was the Discrimination in Medical Settings (DMS) Scale (Peek et al., 2011), which includes seven items scored on a Likert scale (1 = never, 2 = rarely, 3 = sometimes, 4 = most of the time, 5 = always). The DMS is a healthcare-specific modification of the Everyday Discrimination Scale (Williams et al., 1997) that was included in the social determinants of health survey, which launched in November 2021. The instrument questions are: “The next statements describe how others may treat you. How often do any of these happen to you when you go to a doctor’s office or other health care provider? (1) You are treated with less courtesy than other people, (2) You are treated with less respect than other people, (3) You receive poorer service than others, (4) A doctor or nurse acts if he or she thinks you are not smart, (5) A doctor or nurse acts as if he or she is afraid of you, (6) A doctor or nurse acts as if he or she is better than you, and (7) You feel like a doctor or nurse is not listening to what you were saying.” For each of these respective questions, we defined two response cutoffs: first, having experienced “any” healthcare discrimination (categorizing the answers as rarely, sometimes, most of the time, always vs. never) and, second, “frequent” healthcare discrimination (answers sometimes, most of the time, always vs. rarely, never). Overall experience of any and frequent healthcare discrimination was calculated among those who responded to all DMS items and was defined as experiencing any and frequent healthcare discrimination in at least one domain.

Covariates for this analysis included history of cigarette use, sexual orientation, gender, race and ethnicity, income, education, body mass index (BMI), and age. Sexual orientation (straight, lesbian/gay/bisexual, other, prefer not to say/unknown), gender (woman, other/not reported), race and ethnicity (Hispanic, Latino, or Spanish; Non-Hispanic White; Non-Hispanic Black, African American, or African; Non-Hispanic Other, not reported/unknown), education (no high school completion, high school completion, some college, at least college degree, unknown), and income (<$25,000, $25,000–49,999, $50,000-$99,999, ≥$100,000, prefer not to say/unknown) were self-reported as part of the basics survey of demographic information. The “Other” race and ethnicity category includes American Indian or Alaska Native, Middle Eastern or North African, and Native Hawaiian or other Pacific Islander. The “Other” gender category includes genderqueer, genderfluid, gender variant, two-spirit, questioning or unsure, or none of these. History of cigarette use (smoked 100 + cigarettes in life yes/no) was measured in the lifestyle survey. Select physical measurements, including BMI (<25 kg/m^2^, 25–30 kg/m^2,^ >30 kg/m^2^, unknown), were measured at the time of enrollment. Of note, the time of BMI measurement and the basics and lifestyle survey completion relative to the time of social determinants of health survey completion varied based on time of participant enrollment, as the *All of Us Research Program* launched in 2018, but the social determinants of health survey was not launched until 2021.

### 2.3 Statistical analysis

While some of the *All of Us* data are publically available, access to some data and analysis platforms is restricted. We analyzed the data for our study in the Controlled Tier of the *All of Us* Researcher Workbench. We describe demographic sample characteristics as counts, frequencies, and means ± standard deviations (SD). We describe the proportion of participants in the overall sample experiencing any discrimination in any category as well as the percentage of participants who report sometimes or more frequent healthcare discrimination in any category. We conducted logistic regression models to assess the associations of gynecologic cancer type with healthcare discrimination (both “any” and “frequent” definitions). Odds ratio and 95 % confidence intervals (CIs) are reported. Multivariate models were adjusted for the covariates described above (confounders identified *a priori* and available in the dataset). Some categories were collapsed to ensure all reported cells included > 20 participants to protect participant privacy as per *All of Us* guidelines. Analyses were conducted using R in the *All of Us* Researcher Workbench (Wasserstein & Lazar, 2016).

## 3 Results

A total of 2,195 *All of Us* participants with a history of primary gynecologic cancer also responded to at least one item of the DMS survey and were included in our sample. The most common cancer reported in this sample was cervical cancer (45.6 %), followed by endometrial/uterine cancer (26.4 %), ovarian/fallopian tube cancer (19.1 %), more than one cancer (7.6 %), and vaginal or vulvar cancer (1.4 %) (Table 1). Of those with multiple cancer types reported, the most frequently reported combinations were ovarian and uterine, cervical and uterine, and ovarian and cervical. The majority (95.7 %) identified as a woman. The median age of participants was 63.8 years (range 22.7–93.6). Most (88.9 %) of participants identified as straight, 3.5 % identified as bisexual, 2.5 % as lesbian or gay, 1.0 % as another sexual orientation, and 4.1 % preferred not to answer or the response was missing. Most (81.5 %) identified as non-Hispanic White, 6.3 % identified as Hispanic, Latino, or Spanish, 4.9 % identified as non-Hispanic Black, African American, or African, and 3.2 % identified as another non-Hispanic identity. About half of participants had a college degree (54.8 %); 38.5 % were working at the time of survey completion; and 35.6 % were retired.

**Table 1:** Sociodemographic data from 2023 and prior *All of Us* participants with a history of gynecologic cancer (n = 2,195).

**MEASURES**	**n**	**%**
**Primary Cancer Site**		
Cervix	1,001	45.6 %
Endometrium/Uterus	579	26.4 %
Ovary/Fallopian Tube	419	19.1 %
Vagina/Vulva	30	1.4 %
More than One	166	7.6 %

**Gender**		
Woman	2,101	95.7 %
Male, Other, not reported	94	4.3 %

**Race and Ethnicity**		
Hispanic, Latino, or Spanish	139	6.3 %
Non-Hispanic White	1,790	81.5 %
Non-Hispanic Black, African American, or African	107	4.9 %
Non-Hispanic Other	70	3.2 %
Not reported/unknown	89	4.1 %

**Body mass index (BMI, kg/m^2^)**		
<25	505	23.0 %
25-30	423	19.3 %
>30	837	38.1 %
Unknown	430	19.6 %

**Education**		
No high school completion	54	2.5 %
High school completion	228	10.4 %
Some college	646	29.4 %
At least college degree	1182	54.8 %
Not reported/unknown	85	3.9 %

**Employment Status**		
Not Working	490	22.3 %
Working	846	38.5 %
Retired	782	35.6 %
Not reported/unknown	77	3.5 %

**Sexual Orientation**		
Straight	1,952	88.9 %
Bisexual	76	3.5 %
Lesbian/gay	54	2.5 %
None of the above, other sexual orientation	22	1.0 %
Prefer not to answer / Missing	91	4.1 %

**Partner Status**		
Divorced	452	21.0 %
Living with a partner	100	4.6 %
Married	1057	8.8 %
Never Married	264	12.0 %
Separated	46	2.1 %
Widowed	194	8.8 %
Not reported/unknown	82	3.7 %

**Household Income**		
<$25,000	384	17.5 %
$25,000–49,999	390	17.8 %
$50,000-$99,999	580	26.4 %
≥$100,000	547	24.9 %
Prefer not to say / Unknown	294	13.4 %

**Smoked 100 + cigarettes in life**		
No	1134	51.7 %
Yes	1025	46.7 %
Not reported/unknown	36	1.6 %
	**Median**	**Range**
**Age**	63.8	22.7–93.6

“Other” had to be used in some variables for small number reasons to protect participant privacy per *All of Us* guidelines. Category for “Other” Race and Ethnicity created for small numbers reasons and includes American Indian or Alaska Native, Middle Eastern or North African, and Native Hawaiian or other Pacific Islander. “Other” Gender includes genderqueer, genderfluid, gender variant, two-spirit, questioning or unsure, or none of these.

**Fig. 1:**
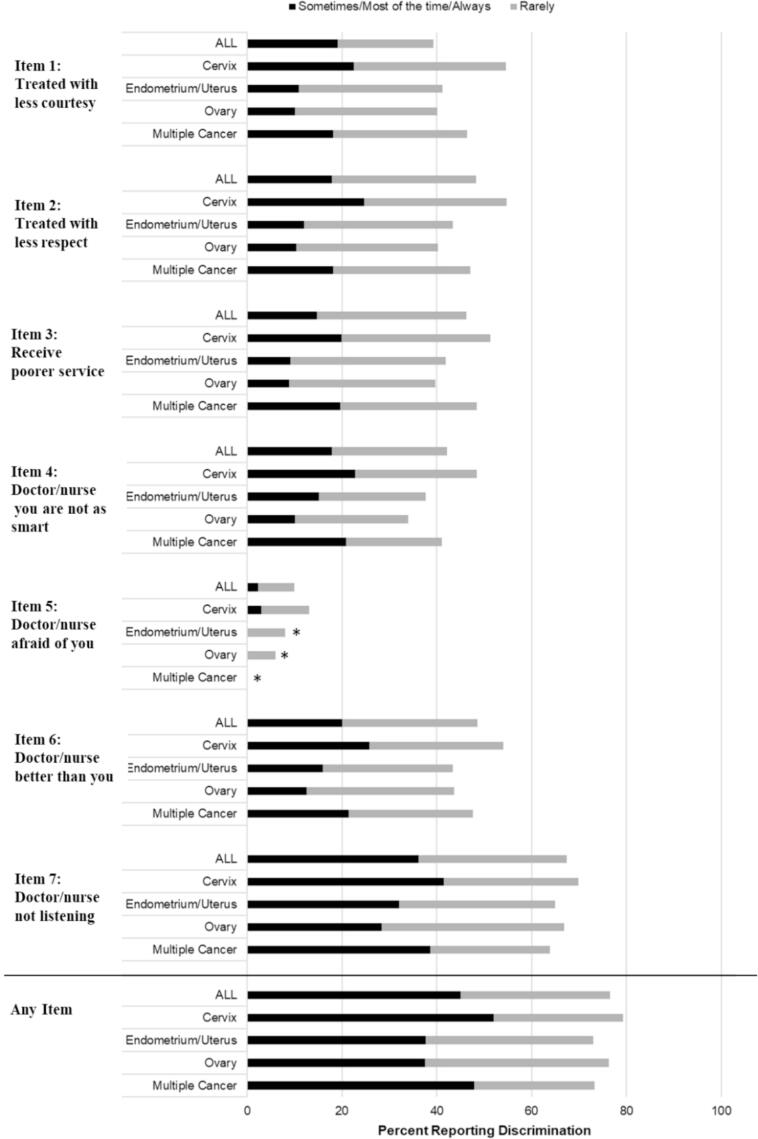
**Healthcare discrimination items by cancer type.** * Data suppressed because of small numbers per *All of Us* policy.

A total of 2,098 participants (95.6 %) answered all DSM items and were included in the composite variables “any” vs. none and “frequent” vs. infrequent healthcare discrimination. In this study population of individuals with a history of gynecologic cancer, 76.5 % reported any experience of healthcare discrimination, and 45.0 % reported frequently experiencing healthcare discrimination (Table 2). Across cancer types and cutoffs, “You feel like a doctor or nurse is not listening to what you were saying” was the most frequently reported experience, and “A doctor or nurse acts as if he or she is afraid of you” was the least frequently reported experience (Fig. 1). Participants with a history of cervical cancer most frequently reported experiencing each individual scale item.

**Table 2:** Experience of healthcare discrimination by cancer type in the *All of Us* cohort.

**Healthcare Discrimination Measure**	**N (%)**	**Odds Ratio** **(95 % CI)**	**Adjusted Odds Ratio** (95 % CI)***
Any Healthcare discrimination vs. Never*			
All (excluding Vagina/vulva+)	1606 (76.5)		
*By gynecologic cancer type*			
Cervix	770 (79.2)	1.18 (0.90, 1.56)	0.93 (0.69, 1.25)
Endometrium/Uterus	409 (73.0)	0.84 (0.63, 1.13)	0.89 (0.65, 1.21)
Ovary/Fallopian Tube	309 (76.3)	Ref.	Ref.
Multiple	118 (73.3)	0.85 (0.56, 1.30)	0.74 (0.48, 1.15)

Frequent healthcare discrimination vs. Never/Rarely**			
All (excluding Vagina/vulva+)	945 (45.0)		
*By gynecologic cancer type*			
Cervix	505 (52.0)	1.80 (1.42, 2.28)	1.35 (1.04, 1.75)
Endometrium/Uterus	211 (37.7)	1.01 (0.77, 1.31)	1.15 (0.87, 1.53)
Ovary/Fallopian Tube	152 (37.5)	Ref.	Ref.
Multiple	77 (47.8)	1.53 (1.05, 2.21)	1.35 (0.91, 2.00)

* “Rarely, sometimes, most of the time, or always” reported for any measure.

** “Sometimes, most of the time, or always” reported for any measure.

*** Adjusted for age, race and ethnicity, income, education, cigarette use, sexual orientation, and body mass index. Vagina and vulva are not included because of small sample sizes.

+ Rows with fewer than 20 participants cannot be reported to protect participant privacy per All of Us policy.

Experience of healthcare discrimination differed by gynecologic cancer type. Participants with a history of cervical cancer most often reported experiencing each individual DSM item (Fig. 1) as well as composite “any” and “frequent” healthcare discrimination (Table 2). This difference was largest for frequent healthcare discrimination, where 52 % of people with a history of cervical cancer reported frequently experiencing healthcare discrimination compared to 37.5 % of people with a history of ovarian cancer (OR: 1.80, 95 % CI: 1.42, 2.28). In the adjusted model, the effect size was attenuated (OR: 1.35, 95 % CI: 1.04, 1.75). Experiences of frequent, but not any, healthcare discrimination were also more common for those with a history of multiple gynecologic malignancies compared to those with a history of ovarian cancer, with 47.8 % and 37.5 % reporting frequent healthcare discrimination, respectively (unadjusted OR: 1.53, 95 % CI: 1.05, 2.21 and adjusted OR: 1.35, 95 % CI: 0.91, 2.00).

## 4 Discussion

We examined the experience of healthcare discrimination among people with a history of gynecologic cancer and associations of gynecologic cancer type with reported experience of healthcare discrimination. More than three quarters of participants reported experiencing some healthcare discrimination, and healthcare discrimination was reported across all gynecologic cancer types. Those with cervical cancer and, to a lesser extent, multiple gynecologic cancers reported more often experiencing healthcare discrimination, and this trend was generally consistent across individual item measures. There were greater differences by cancer type observed in the experience of frequent healthcare discrimination (as compared with the any healthcare discrimination measure), suggesting that the difference is concentrated among those reporting highest levels of discrimination.

We observed a greater frequency of participants with a history of cervical cancer reporting discrimination than other cancer types, even after adjustment for other variables associated with differences in cervical cancer risk and discrimination including race and income. One potential contributor may be cancer-related stigma, which has been shown to negatively affect cancer screening uptake and care engagement (Carter-Harris, 2015, Edelen et al., 2014, Marlow and Wardle, 2014, Meacham et al., 2016, Vrinten et al., 2019). Cervical cancer in particular is commonly stigmatized because it is linked to HPV and sexual health and viewed as indicative of a person’s behavior (Peterson et al., 2021). It is therefore possible that those with a history of cervical cancer could report more healthcare discrimination because of stigmatization from their cancer teams or other clinicians. Because we do not know when the healthcare discrimination occurred, it is possible that healthcare discrimination was experienced prior to their cancer diagnosis and resulted in reduced trust in counseling and less access to HPV vaccination, cervical cancer screening, and other resources that could have prevented cervical cancer (Alfaro et al., 2021, Tracy et al., 2010). A less equitable healthcare system and increased barriers to care contribute more to incidence of preventable cancers than to cancers with fewer screening options or early detection methods, such as ovarian cancer (Alfaro et al., 2021, Stewart et al., 2019, Tracy et al., 2010). Future research should assess when individuals are experiencing healthcare discrimination.

Individuals categorized as having multiple gynecologic cancers also reported more frequent experiences of healthcare discrimination. This may be a result of compounding stigma from having multiple cancers and/or inequitable healthcare system experiences prior to diagnosis that could have put these individuals at greater risk for multiple cancers. It is also possible that, instead of observing a true multiple cancer effect, we are observing a partial cervical cancer effect as more than half of individuals with multiple cancers had cervical cancer as one of their cancer types.

With the focus on discrimination in medical settings specifically, this study contributes to our understanding of discrimination among individuals with gynecologic cancers, which to date was predominantly comprised of work investigating cancer diagnoses and screening as well as everyday discrimination, heterosexism, and racism, among which systemic review found negative effects of racism across cancer screening, treatment, and survivorship (Agénor et al., 2015, Hirschey et al., 2023, Jacobs et al., 2013, Mullins et al., 2019, Tabaac et al., 2019, Washington and Randall, 2023). Further, our findings suggest that, in addition to previously observed differences in reported healthcare discrimination among people with a history of cancer by disease status, income, and other reasons from multiple levels of the healthcare system (Caston et al., 2022), individuals with specific cancers may experience more discrimination in healthcare settings than others.

Strengths of this study include the use of a previously validated measure for healthcare discrimination and large sample size relative to the existing literature on discrimination experiences in individuals with gynecologic cancer. Limitations include fewer participants of color compared to non-Hispanic White individuals despite intentional recruitment. While we controlled for race and ethnicity, these categories are extremely broad and do not intricately capture the differences in social and structural barriers individuals and communities experience, leaving room for residual confounding. Similarly, we were unable to separate individual gender identities from women and other/missing. In addition, our outcomes may have been impacted by the way in which cancers were reported. The dataset included more individuals with cervical cancer than would be expected given United States gynecologic cancer prevalence and incidence. This difference came mostly from the self-report data, where the largest category of self-reported cancers were cervical cancers. It is possible that precancerous cervical lesions were reported as cancers by self-report or that this is the true prevalence in this intentional sample of people underrepresented in medical research. We are also unable to assess if those who experienced discrimination were more, less, or equally likely to respond to the discrimination in medical settings measure. In the overall *All of Us* sample, some participants who would be expected to report higher levels of discrimination—such as those who identified as Black and Hispanic, used the Spanish version of the survey, and had less education—had higher percentages of non-response (Tesfaye et al., 2024), but it is also possible that those who felt moved to share their experiences with healthcare discrimination were more likely to respond. There has also been investigation into the challenges of comparing the Everyday Discrimination Scale accross social groups (Bastos & Harnois, 2020); while we control for different social groups in our model, it is still possible that people with different cancers understand the discrimination questions differently and/or have different discrimination experiences that impact their responses. Finally, while it would have been preferable to analyze and present the data for each response category separately (e.g. “sometimes” separate from “most of the time” and “always”), cells with fewer than 20 participants may not be reported to protect participant privacy per the *All of Us* privacy policy*.*

## 5 Conclusions

Experience of discrimination in the healthcare setting was reported by individuals across gynecologic cancer types. Individuals with a history of cervical cancer or multiple gynecologic malignancies reported more frequent healthcare discrimination than those with other gynecologic cancers, even after adjustment for demographic factors associated with discrimination. Future research should examine in detail how healthcare discrimination is manifesting and how it impacts cancer care and outcomes. It is crucial for gynecologic oncologists and other healthcare providers involved in their care to acknowledge the potential for healthcare discrimination among patients with gynecologic cancer diagnoses and recognize how such experiences may create additional barriers to care.

## Funding

This research was supported in part by the United States National Institutes of Health (NIH, P30CA77598, UM1TR004405). The content is solely the responsibility of the authors and does not necessarily represent the official views of the National Institutes of Health. A.C.D. was supported by the NIH MSTP grant T32 GM008244 and NIH F30 grant 1F30MD019959-01. The data were presented in part as a poster at the 2024 Society of Gynecologic Oncology Annual Meeting in San Diego, CA.

## CRediT authorship contribution statement

**Allison C. Dona:** Writing – review & editing, Writing – original draft, Methodology, Conceptualization. **Patricia I. Jewett:** Writing – review & editing, Methodology, Formal analysis, Conceptualization. **Sarah Davidson:** Writing – review & editing. **Deanna Teoh:** Writing – review & editing, Conceptualization. **Rachel I. Vogel:** Writing – review & editing, Visualization, Supervision, Methodology, Formal analysis, Conceptualization.

## Declaration of competing interest

The authors declare that they have no known competing financial interests or personal relationships that could have appeared to influence the work reported in this paper.
